# Inequity in dialysis related practices and outcomes in Aotearoa/New Zealand: a Kaupapa Māori analysis

**DOI:** 10.1186/s12939-018-0737-9

**Published:** 2018-02-20

**Authors:** Tania Huria, Suetonia Palmer, Lutz Beckert, Jonathan Williman, Suzanne Pitama

**Affiliations:** 1 Māori and Indigenous Health Institute, University of Otago Christchurch, 2 Riccarton Ave, Christchurch, 8140 New Zealand; 2 Department of Medicine, University of Otago Christchurch, Christchurch, New Zealand; 3 Department of Population Health, University of Otago Christchurch, Christchurch, New Zealand

**Keywords:** Indigenous, Māori, Disparities, Equity, Dialysis

## Abstract

**Background:**

In Aotearoa/New Zealand, Māori, as the indigenous people, experience chronic kidney disease at three times the rate of non-Māori, non-Pacific New Zealanders. Māori commence dialysis treatment for end-stage kidney disease at three times the rate of New Zealand European adults. To examine for evidence of inequity in dialysis-related incidence, treatment practices, and survival according to indigeneity in Aotearoa/New Zealand, utilising a Kaupapa Māori approach.

**Methods:**

We conducted a retrospective cohort study involving adults who commenced treatment for end-stage kidney disease in Aotearoa/New Zealand between 2002 and 2011. We extracted data from the Australian and New Zealand Dialysis and Transplant Registry (ANZDATA) linked to the New Zealand National Health Index (NHI). Propensity score methods were used to assemble a cohort of 1039 Māori patients matched 1:1 on clinical and socio-demographic characteristics with a cohort of 1026 non-Māori patients. We compared incidence of end-stage kidney disease and treatment practices. Differences in the risks of all-cause mortality during treatment between propensity-matched cohorts were estimated using Cox proportional hazards and generalised linear models.

**Results:**

Non-Māori patients were older, more frequently lived in urban areas (83% versus 67% [standardised difference 0.38]) and bore less socioeconomic deprivation (36% living in highest decile areas versus 14% [0.53]). Fewer non-Māori patients had diabetes (35% versus 69%, [− 0.72]) as a cause of kidney failure. Non-Māori patients were more frequently treated with peritoneal dialysis (34% versus 29% [0.11]), received a pre-emptive kidney transplant (4% vs 1% [0.19]), and were referred to specialist care < 3 months before treatment (25% vs 19% [0.15]) than Māori patients. Fewer non-Māori started dialysis with a non-tunnelled dialysis vascular catheter (43% versus 47% [− 0.08]). The indigenous-age standardised incidence rate ratio for non-Māori commencing renal replacement therapy in 2011 was 0.50 (95% CI, 0.40–0.61) compared with Māori.

Propensity score matching generated cohorts with similar characteristics, although non-Māori less frequently started dialysis with a non-tunnelled venous catheter (30% versus 47% [− 0.35]) or lived remotely (3% versus 14% [− 0.50]). In matched cohorts, non-Māori experienced lower all-cause mortality at 5 yr. after commencement of treatment (risk ratio 0.78, 95% CI 0.72–0.84). New Zealand European patients experienced lower mortality than Māori patients in indigenous age-standardised analyses (age-standardised mortality rate ratio 0.58, 95% CI 0.51–0.67).

**Conclusions:**

Non-Māori patients are treated with temporary dialysis vascular access less often than Māori, and experience longer life expectancy with dialysis, even when socioeconomic, demographic, and geographical factors are equivalent. Based on these disparities, health services should monitor and address inequitable treatment practices and outcomes in end-stage kidney disease care.

## Background

Chronic kidney disease disproportionally impacts indigenous peoples [1–3]. Inequitable health outcomes for indigenous peoples related to kidney disease and other long-term conditions have not been adequately explained by existing epidemiological approaches. Inequities in health outcomes have persisted despite considerable research and policy efforts, and are entrenched [2, 4]. An accepted explanation for inequitable outcomes is that increased rates of kidney disease for indigenous peoples are attributable to higher rates of poverty, diabetes, hypertension, and cardiovascular disease [5, 6]. Studies have also identified socioeconomic deprivation and remote living as putative factors contributing to lower life expectancy and chronic disease risk [1, 7]. It is essential that research methodologies are employed to inform understanding of inequity in the setting of chronic disease [8].

In Aotearoa/New Zealand, Māori, as the indigenous people, experience chronic kidney disease at three times the rate of non-Māori, non-Pacific New Zealanders [3]. Māori commence dialysis treatment for end-stage kidney disease at three times the rate of New Zealand European adults [1, 2, 9, 10]. For example, in 2015, New Zealand European adults incurred an incidence of dialysis nearly four times lower that of Māori adults (72 versus 266 per million population) [11]. It is assumed that disparate treatment practices are driven by comorbidity and socio-economic factors [12]. In contrast to the decreasing incidence of dialysis in non-indigenous populations globally, dialysis rates for Māori have not declined over time [1, 2, 9, 10].

Existing scientific methodologies frame inequity as a deficit present within indigenous peoples and, as such, may not enable researchers and healthcare providers to observe or consider how systemic advantages are sustained for non-indigenous populations within health services [13]. Accordingly, it is possible that non-indigenous research approaches may impede development of policy and health service responses to inequity and prevent health gains for Māori [14]. Given the sustained inequity in health outcomes for Aotearoa/New Zealand, we applied indigenous methodologies to explore for potential sources of inequitable treatment practices and outcomes for Māori with end-stage kidney disease [14, 15].

## Methods

### Indigenous approach

We employed a Kaupapa Māori approach to include the broader political context for research that involves Māori [15]. A Kaupapa Māori approach enabled research practices within this study to enact the principles of the Treaty of Waitangi. This foundational document of Aotearoa/New Zealand from 1840 defines the constitutional relationship between the Treaty partners, the British Crown and iwi, the governing structures for Māori as the indigenous peoples of Aotearoa/New Zealand [16]. In particular, the research process incorporates Article 3 of the Treaty, that charges the British Crown with the responsibility to provide equity – and therefore health equity – for Māori, as a human right. In recognition of the Treaty partners and this obligation in the study design, we defined Māori and non-Māori as the comparative study cohorts in the primary analysis. We aligned the study methodology with the United Nation Declaration on the Rights of indigenous peoples, and thus included Māori as patients who self-identified as Māori and who were resident in Aotearoa/New Zealand [17].

The Kaupapa Māori approach included utilisation of indigeneity as a ‘principle’ within the analysis. Indigeneity, when considered as a principle, enabled being Māori to be analysed as marker of risk (for example, risk of exposure to colonisation, poverty, and institutional racism), as opposed to utilising indigeneity as a variable, expressed as a determinant of health practices or outcomes in itself [13]. The Kaupapa Māori methodology incorporated indigenous age standardisation, that utilised the 2013 Māori census population as the reference standardised population, to account for the different age structures of the Māori and non-Māori populations [18, 19].

### Study population

We included all adults (aged ≥18 years) who commenced renal replacement therapy (dialysis or kidney transplantation) as first treatment for end stage kidney disease living in Aotearoa/New Zealand between 1 January 2002 and 31 December 2011 [20]. Patients were censored at the end of the study (31 December 2011), death, or loss to follow up.

### Data collection

We extracted data from ANZDATA and linked these data with the New Zealand National Health Index (NHI) to identify prioritised ethnicity categories and include deprivation and rurality information [21, 22]. Socio-demographic and clinical variables were extracted including: age, gender, ethnicity (labelled in ANZDATA as “racial origin”), postcode, weight, height, medical comorbidities, primary cause of kidney disease (diabetes, hypertension/ischaemic heart disease, glomerulonephritis, polycystic kidney disease, obstruction, and other), late referral to specialist nephrology services (referred < 3 months before first treatment), dialysis vascular access type (arteriovenous fistula, arteriovenous graft, or central venous catheter [tunnelled or non-tunnelled]), and initial treatment modality at the commencement of renal replacement therapy (haemodialysis, peritoneal dialysis, or kidney transplantation).

We categorised rurality as rural (no or low urban influence), independent urban (minimal major urban dependence), and urban (major urban area) [23]. Socioeconomic deprivation indices were drawn from the NZDep2013 [21]. The NZDep2013 deprivation score combines census data relating to income, home ownership, employment, qualifications, family structure, housing, access to transport and communications. A deprivation score is assigned to each meshblock (the smallest geographical area defined by Statistics New Zealand including a population of 60–100 people) [24, 25]. Deprivation scores are expressed in deciles [26]. A score of one represents areas with the least deprivation and ten the areas with the most. Indigeneity was self- identified within the National Health Index, which aligned with New Zealand ethnicity data collection protocols [18, 27]. Denominator populations were defined for Māori and non-Māori as the cohort-specific estimated New Zealand resident populations counted on 30 June of the corresponding year [18].

### Statistical analysis

Sociodemographic and clinical characteristics at baseline were summarised as mean and standard deviation for continuous variables and number and proportion for dichotomous variables. Summary baseline characteristics were compared between Māori and non-Māori patients using standardised differences calculated by the Austin formula [28]. Standardised differences of 0.2, 0.5, and 0.8 were considered to represent small, moderate, and large differences between cohorts, respectively.

The primary outcome for the analysis was all-cause mortality. Secondary outcomes were the modality of first treatment and dialysis vascular access type for those commencing haemodialysis.

Propensity score matching was used to assemble Māori and non-Māori cohorts with similar clinical and sociodemographic characteristics to explore differences in the primary and secondary outcomes [28, 29]. Propensity scores were obtained as the predicted probabilities of a logistic regression that included variables that are associated with mortality and dialysis modality. These included: age, gender, body mass index, deprivation score, smoking status and diabetes. Māori patients were matched 1-to-1 with non-Māori patients using a nearest neighbour algorithm [30]. We assessed the balance of characteristics between the matched cohorts before and after matching, expressed as a standardised difference.

We used a Cox proportional hazards model (Breslow method) to evaluate the association of indigeneity with all-cause mortality after commencing dialysis within the propensity score-matched indigenous (Māori) and non-indigenous (non-Māori) cohorts. There was evidence that the hazard for all-cause mortality was not proportional, so we additionally calculated risk ratios of all-cause mortality at 1, 3, and 5 years in matched cohorts (Table 2). As there were differences between the proportion of patients living in urban or rural areas between the propensity-score matched cohorts, all regression analysis of propensity-score matched cohorts were also adjusted for rurality. We used a reverse Kaplan-Meier method to calculate median follow up time [31].

Sensitivity analyses were done within the non-Māori cohort, identifying separate New Zealand European and Pacific cohorts. We then used direct age standardisation within each cohort (Māori, New Zealand European, and Pacific), incorporating Māori as the reference category, to compare age-standardised risk of mortality between groups [19].

Analyses were performed using STATA version 13. The study was approved by the University of Otago Ethics B committee (HD14/27).

## Results

### Study population

Overall, 4781 patients commenced treatment within the study period. Of these, nine patients were excluded due to record duplication. Accordingly, 1459 Māori adults and 3312 non-Māori adults were included in the analysis.

At commencement of treatment with dialysis or transplantation, non-Māori were older (58 ± 15 years versus 56 ± 12 years [standardised difference 0.15]), and were less likely to smoke (12% versus 26% [standardised difference − 0.36]), have diabetes as cause of kidney disease (35% versus 69% [standardised difference − 0.72]), and had a lower body mass index (28 ± 7 kg/m^2^ versus 33 ± 8 kg/m^2^ [standardised difference − 0.67]) (Table 1). Non-Māori more frequently lived in an urban setting (83% versus 67% [standardized difference 0.83]) and less frequently lived in areas with socioeconomic deprivation (decile 9 and 10: 31% versus 59% [standardised difference − 0.59]). After propensity-score matching, the large standardized differences between cohorts for many baseline characteristics were reduced to small standardized differences including: age (0.03), sex (0.04), NZDep13 (0.00), current smoking history (0.05), and diabetes as primary renal disease (− 0.09). Despite propensity score matching, non-Māori less frequently lived in a rural area (− 0.52).

**Table 1:** Baseline characteristics of incident dialysis patients in Aotearoa/New Zealand, according to indigenous status

	Whole Cohort			Cohort after propensity matching		
	Non-Māori *n* = 3312	Māori *n* = 1459	Standardised Difference	Non-Māori *n* = 1026	Māori *n* = 1039	Standardised Difference
Age, years^a^	58 (15)	56 (12)	0.15	55 (13)	55 (12)	0.03
Women^a^	1310 (40)	601 (41)	−0.02	564 (39)	595 (41)	−0.04
Domicile Code						
Urban	2728 (83)	981 (67)	0.38	1270 (88)	974 (67)	0.52
Semi-urban	342 (10)	268 (18)	−0.23	130 (9)	264 (18)	−0.27
Rural	234 (7)	206 (14)	−0.23	45 (3)	206 (14)	−0.50
Deprivation score^a^						
1 to 5	1184 (36)	206 (14)	0.53	202 (14)	205 (14)	0.00
6 to 8	1086 (33)	396 (27)	0.13	426 (29)	393 (27)	0.04
9 to 10	1034 (31)	854 (59)	−0.59	817 (57)	847 (59)	−0.04
Smoking status^a^						
Never	1663 (50)	406 (28)	0.46	441 (31)	402 (28)	0.07
Former	1243 (38)	679 (47)	−0.18	652 (45)	671 (46)	−0.02
Current	404 (12)	373 (26)	−0.36	352 (24)	372 (26)	−0.05
Laboratory variables						
Serum creatinine μmol/L	721 (333)	783 (350)	−0.18	753 (322)	782 (350)	−0.09
Haemoglobin, g/L	110 (17)	108 (17)	0.12	109 (17)	108 (17)	0.06
Body mass index kg/m^2a^	28 (7)	33 (8)	−0.67	33 (9)	33 (8)	0.00
Primary renal disease						
Diabetes^a^	1143 (35)	1010 (69)	−0.72	940 (65)	1001 (69)	−0.09
Hypertension/ischaemic	446 (14)	80 (5)	0.31	88 (6)	79 (5)	0.04
Glomerulonephritis	941 (28)	228 (16)	0.29	250 (17)	228 (16)	0.03
Polycystic kidney disease	241 (7)	23 (2)	0.24	37 (3)	22 (2)	0.06
Urological	146 (4)	25 (2)	012	35 (2)	25 (2)	0.00
Other	395 (12)	93 (6)	0.21	95 (7)	90 (6)	0.04
Comorbid medical conditions						
Diabetes						
Type 1	116 (4)	27 (2)	0.12	69 (5)	27 (2)	0.16
Type 2	1241 (37)	1042 (72)	−0.75	1014 (70)	1033 (71)	−0.02
Coronary artery disease	875 (26)	410 (28)	−0.05	415 (29)	402 (28)	0.02
Peripheral vascular disease	458 (14)	281 (19)	−0.14	260 (18)	273 (19)	−0.03
Cerebrovascular disease	373 (11)	156 (11)	0	181 (13)	153 (11)	0.06
Chronic lung disease	354 (11)	275 (19)	−0.23	196 (14)	273 (19)	−0.14
Cancer	692 (21)	204 (14)	0.19	192 (13)	203 (14)	−0.03
Year of starting renal replacement therapy						
2002–2003	609 (18)	282 (19)	−0.03	260 (18)	274 (19)	−0.03
2004–2006	955 (29)	430 (30)	−0.02	366 (25)	393 (27)	−0.05
2007–2008	643 (19)	289 (20)	−0.03	366 (25)	413 (29)	−0.09
2009–2011	1105 (34)	458 (31)	0.06	453 (31)	365 (25)	0.13

Data are presented as number (proportion) or mean (SD). Standardised differences of 0.2, 0.5 and 0.8 can be considered to represent small, medium and large differences [25, 38]. Deprivation based on NZDep13 which is a socio economic deprivation index used in Aotearoa/New Zealand where 10 = lowest decile (most deprived) and 1 = highest decile (least deprived) [19]. Urban is defined as most urbanised areas of Aotearoa/New Zealand, semi-urban is defined as towns and settlements without significant dependence on main urban centers, and rural is defined as areas with low urban influence [21]. ^a^ Variables included in the propensity score modeling

### Incidence of renal replacement therapy

**Fig. 1:**
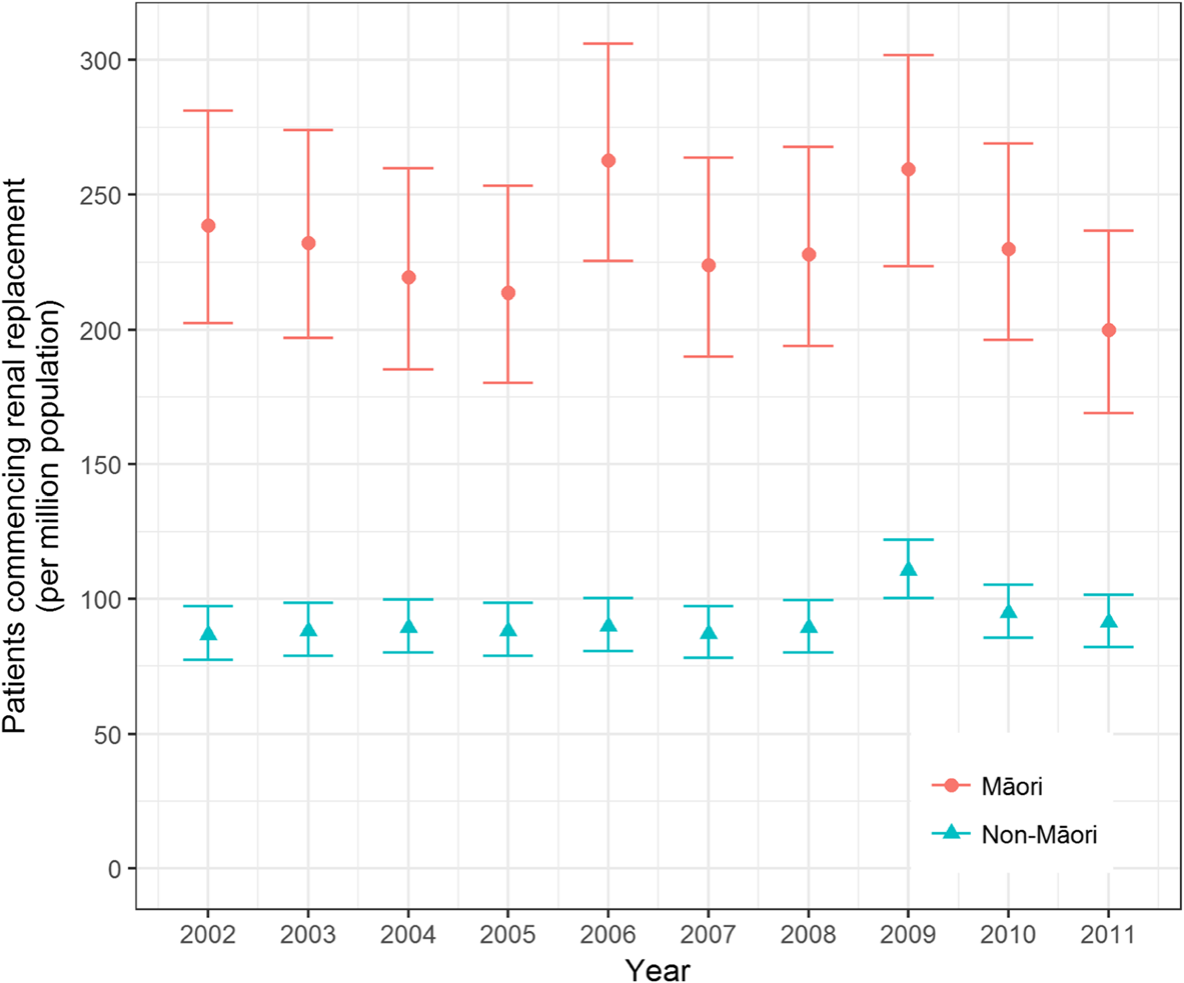
Incidence rates for commencing renal replacement therapy

The annual incidence of commencing renal replacement therapy per million population between 2002 and 2011 is shown in Fig. 1 according to indigeneity. The indigenous-age standardised incidence rate ratio for non-Māori commencing renal replacement therapy in 2011 was 0.50 (95% CI, 0.40–0.61) compared with Māori.

### Primary outcome: All-cause mortality

Patients were followed up for a median of 57.0 months after commencement of dialysis or transplantation. There were 2284 deaths (1465 non-Māori and 847 Māori). After propensity score matching, there were 1186 deaths during follow up including 513 for non-Māori and 673 for Māori.

**Fig. 2:**
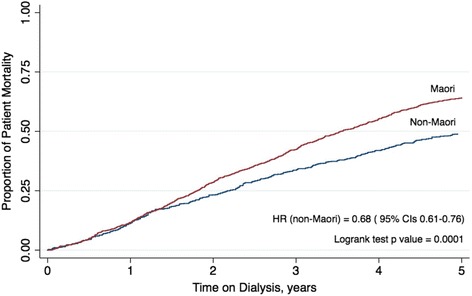
Kaplan Meier of propensity score matched cohort of mortality by indigeneity and the number of years on renal replacement therapy

In unadjusted analysis, the risk of all-cause mortality was lower for non-Māori (unadjusted hazard ratio [HR] 0.82, 95% CI, 0.75–0.89). In survival analysis comparing the propensity score matched cohorts, non-Māori had a lower risk of mortality (HR 0.68, 95% CI 0.61–0.76) (Fig. 2). There was no evidence of a different risk of all-cause mortality between non-Māori and Māori at 1 year after starting therapy) (Table 2). Non-Māori experienced a lower risk of all-cause mortality than Māori at 3 and 5 years after commencing treatment and adjusted for rurality.

**Table 2:** All-cause mortality at 1, 3, and 5 years after commencing treatment for end-stage kidney disease according to indigenous status

Indigenous status, deaths (%)	Years since commencement of dialysis		
	One	Three	Five
Māori (*N* = 1039)	122 (11.7)	444 (42.7)	673 (64.8)
Non-Māori (*N* = 1026)	120 (11.7)	353 (34.4)	513 (50.0)
Risk ratio^a^ (95% CI)	1.07 (0.83–1.38)	0.83 (0.74–0.93)	0.78 (0.72–0.84)

^a^Non-Māori vs Māori. Risk ratios are adjusted for rurality

### Secondary outcomes: Treatment practices

Treatment practices for renal replacement therapy are shown in Table 3. Non-Māori were less frequently referred late to specialist renal services (19% versus 25% [standardised difference − 0.15]). Non-Māori were more frequently treated with peritoneal dialysis (34% versus 29% [0.11]) or access pre-emptive kidney transplantation (4% versus 1% [0.17]). Fewer non-Māori started dialysis with a non-tunnelled dialysis vascular catheter (43% versus 47% [− 0.08]) and more experienced a functioning arteriovenous fistula at dialysis start (26% versus 23% [standardised difference 0.07]). Despite propensity matching, fewer non-Māori patients commenced dialysis with a non-tunnelled central venous catheter (standardised difference − 0.35) and the standardized difference in peritoneal dialysis as a first treatment modality was reduced to (0.00).

**Table 3:** Baseline treatment practice characteristics

	Whole Cohort					Propensity Matched Cohort				
Treatment Practices	Non-Māori, n (%) *n* = 3312	Māori, n (%) *n* = 1459	Risk ratio	95% CI	Standardised difference	Non-Māori, n (%) *n* = 1026	Māori, n (%) *n* = 1039	Risk Ratio	95% CI	Standardised difference
Modality of first treatment										
Peritoneal Dialysis	1110 (34)	420 (29)	1.16	1.06–1.28	0.11	420 (29)	418 (29)	1.02	0.92–1.13	0
Haemodialysis	2063 (62)	1027 (70)	0.88	0.85–0.92	−0.17	998 (69)	1015 (70)	0.99	0.97–1.00	−0.02
Transplant	139 (4)	12 (1)	5.10	2.84–9.17	0.19	27 (2)	12 (1)	2.28	1.16–4.47	0.08
*Haemodialysis vascular access										
Arteriovenous fistula	468 (26)	206 (23)	1.12	0.97–1.30	0.07	224 (26)	205 (23)	1.12	0.95–1.32	0.07
Arteriovenous graft	21 (1)	18 (2)	0.58	0.31–1.08	−0.08	11 (1)	18 (2)	0.62	0.30–1.31	− 0.08
Tunnelled central venous catheter	546 (30)	247 (28)	1.09	0.96–1.24	0.04	288 (33)	242 (28)	1.22	1.05–1.40	0.11
Non-tunnelled central venous catheter	766 (43)	419 (47)	0.90	0.83–0.99	−0.08	337 (30)	414 (47)	0.83	0.75–0.93	−0.35
Referral practices										
Referral to specialist services within 3 months of starting treatment	620 (19)	371 (25)	0.74	0.65–0.82	−0.15	365 (25)	293 (20)	1.26	1.11–1.43	0.12

*The difference in population sample size between the whole cohort and for vascular access is due to missing data collection of vascular access data at clinical sites. There was *n* = 1801 non-Māori and *n* = 890 Māori with baseline data for dialysis vascular access (and *n* = 860 and *n* = 879 included in propensity score matched cohorts)

### Sensitivity analyses

In sensitivity analysis and to obtain indigenous age-standardization for all-cause mortality, we further disaggregated the non-Māori cohort into New Zealand European (*n* = 1814), Pacific (*n* = 929), and other ethnicities (*n* = 433) (Table 4). Indigenous age-standardization led to an adjusted mortality rate ratio for Māori of 1.72 (95% CI 1.50–1.97) compared with New Zealand European. Pacific patients experienced a higher age-standardized mortality rate ratio than NZ European (1.33, 95% CI 1.16–1.52) but the rate ratio remained lower compared with Māori (1.38 95% CI 1.27–1.51).

**Table 4:** Mortality outcomes during treatment for end-stage kidney disease according to indigenous status in Aotearoa/New Zealand

Ethnicity	Patients, n	Follow-up (person-years)	Deaths, n	Crude mortality rate (per 100 person-years)	Age standardized mortality rate (per 100 person-years)	Crude mortality rate ratio	Indigenous age-standardised mortality rate ratio (95% CI)^a^
Māori	1459	4.84	847	12	8.4	1.32	1.72 (1.50–1.97)
Pacific	929	4.15	389	10.1	6.5	1.11	1.33 (1.16–1.52)
New Zealand European	1814	4.92	810	9.1	4.9	1.00 (ref)	1.00 (ref)

^a^Mortality rates were age standardized to the 2001 Māori census population

## Discussion

A Kaupapa Māori approach to exploring inequity enabled use of best practice ethnicity protocols, incorporated the obligations of the Treaty of Waitangi toward health equity, and adjusted for complex sociodemographic factors and indigenous age standardisation. Using a Kaupapa Māori analysis, this study demonstrates persistent inequity in dialysis incidence, mortality, and treatment practices for patients in Aotearoa/New Zealand. Even when sociodemographic characteristics, comorbidity, and referral practices are equivalent, non-Māori patients less frequently receive non-tunnelled dialysis vascular access when starting dialysis treatment and experience lower mortality risk at 3 and 5 years after starting treatment. These differences in incidence, treatment practices and mortality during treatment for end-stage kidney disease suggest that healthcare systems for dialysis sustain inequitable practices and survival outcomes for Māori. Temporary vascular access is associated with lower survival and increased infection-related morbidity [32]; therefore the lower use of temporary vascular access for non-Māori after controlling for comorbidity to specialist services warrants further scrutiny.

These findings are consistent with observations made by other investigators showing that non-Māori receive higher quality care within New Zealand healthcare services [33], including lower rates of unplanned hospital readmission and death within 30 days [34]. New Zealand European patients are less likely to experience racism and discrimination, factors that are associated with poorer mental and physical health [35]. The better dialysis-related practices and clinical outcomes for non-Māori in this current study are also consistent with recent findings showing that dialysis treatment continues to benefit non-indigenous Australians with greater access to home based dialysis and preferred dialysis modalities than for indigenous Australians [13]. Taken together with the existing literature, our findings add to the growing body of evidence that renal health services in New Zealand advantage non-Māori patients and sustain health inequities.

In practice, policy makers and dialysis services need to consider appropriate interventions to ensure equitable access to quality care for end stage kidney disease. Specifically, health services should ensure that dialysis services provide access to peritoneal dialysis and pre-emptive transplantation for Māori and non-Māori, and early referral to specialist services for patients who require renal replacement therapy. Policy makers and clinicians need to identify effective ways to enable timely permanent dialysis vascular access, and practices that are associated with better outcomes for patients on renal replacement therapy including home based care, longer hours’ dialysis, and kidney transplantation. Further work within renal services is needed to identify interventions that ensure inequitable practices and outcomes including sustainable access to preferred treatment options.

In a previous action research study, changes to health systems at the community and family/whānau, health practitioner, and health service level were identified to address inequity in heart disease management (including systems to support access to hospital appointments, pre-hospital fibrinolytic therapy, and strategy planning for disease prevention) [36]. In dialysis care, and based on the findings in the present study, potential actions might include supporting greater access to permanent vascular access and kidney transplantation, and identifying quality improvement activities to reduce morbidity for dialysis patients. Addressing inequity in healthcare also requires setting expectations that organisations will deliver equity as a measure of quality care, embed health inequity interventions within operating policies, and monitor care quality regularly [37]. In practice, this could include regular monitoring of outcomes for Māori and non-Māori with end-stage kidney disease. In addition, policy-makers and clinicians need to consider effective innovations that advance Māori health outcomes, including community initiated interventions [7, 36–38].

While the strengths of the study included a Kaupapa Māori approach, propensity score matching to account for comorbidities and demographics, a large population dataset (ANZDATA) and indigenous age standardisation, the study has limitations which need to be considered when interpreting the results. First, this is a retrospective study and variables which have been shown in other studies to be associated with Māori and non-Māori life expectancy were not directly accounted for, such as housing [7], education, and income [18]. This may have resulted in residual bias in the results, although the study included the NZDep2013 deprivation score to account for a range of complex socioeconomic factors associated with specific rurality. Second, the study design did not account for time-varying exposure to risk factors for survival such as dialysis-related complications and comorbidity. Third, the use of the National Health Index number to identify participant rurality to identify rurality and deprivation is limited due to the rurality code defaulting to the nearest postal service centre, which may result in bias relating to geographical and deprivation ascertainment. Fourth, neither interactions nor subgroup analysis were used to explore differences in the effect of indigeneity upon mortality in this population. However, there is evidence in the literature that the gap in life-expectancy between Māori and non-Māori in New Zealand differs according to age and smoking status, and these interactions should be included in future research [39]. Finally, this study did not measure access to home based therapy, adjust for hours of dialysis per week, or record or explore treatment adherence.

## Conclusions

This Kaupapa Māori analysis demonstrated that non-Māori patients were less likely to start dialysis, less frequently received non-tunnelled vascular access when starting dialysis, and experienced better survival during treatment for end-stage kidney disease. Non-Māori are advantaged by better treatment outcomes in New Zealand renal care, even when socioeconomic, clinical, and geographical factors are equivalent. Health services and policies need to consider indigeneity as a marker of exposure to risk factors for adverse outcomes in renal care, that warrant action and monitoring, as ways to address disparities in renal care in Aotearoa/New Zealand.

## Abbreviations

ANZDATA: Australia New Zealand Dialysis and Transplantation Registry
CI: Confidence Intervals
HR: Hazard Ratio
NHI: National Health Index
NZDep: New Zealand Index of Deprivation
